# *trans*-Di­aqua­bis­(pyridin-2-yl thio­phen-2-yl ketone-κ^2^*N*,*O*)zinc(II) bis­(tetra­fluorido­borate)

**DOI:** 10.1107/S2414314626010369

**Published:** 2026-10-23

**Authors:** Barry Westcott, Allen G. Oliver

**Affiliations:** a Department of Chemsitry and Biochemistry Central Connecticut State University, 1615 Stanley St New Brtiain CT 06050 USA; b Molecular Structure Facility, 149 Stepan Chemistry Hall, Department of Chemistry and Biochemistry, University of Notre Dame, Notre Dame, IN 46556, USA; Vienna University of Technology, Austria

**Keywords:** crystal structure, zinc, pyridyl ketone, thio­phene, isotypism

## Abstract

The title salt, [Zn(C_10_H_7_NOS)_2_(H_2_O)_2_](BF_4_)_2_, crystallizes isotypically with the Co^II^ analog in the monoclinic space group *P*2_1_/*n*. In the com­plex cation, the central Zn^II^ atom (site symmetry 1) has a pseudo-octa­hedral coordination environment with bonding to pyridyl N [2.0624 (18) Å] and carbonyl O [2.1030 (13) Å] atoms from the 2-thienyl 2-pyridyl ketone ligands. The remaining bonds are to water mol­ecules [2.1598 (16) Å], which form an O—H⋯F hy­dro­gen-bonded layer with BF_4_^−^ counter-ions.

## Abstract 2

The Zn^II^ atom in the title com­plex cation (site symmetry 1) shows a pseudo-octa­hedral coordination by two aqua ligands and two chelating ligands bound through the carbonyl O and pyridyl N atoms, which is consistent with other late first-row transition-metal structures of this ligand with Co^II^, Ni^II^, and Cu^II^.

## Graphical abstract

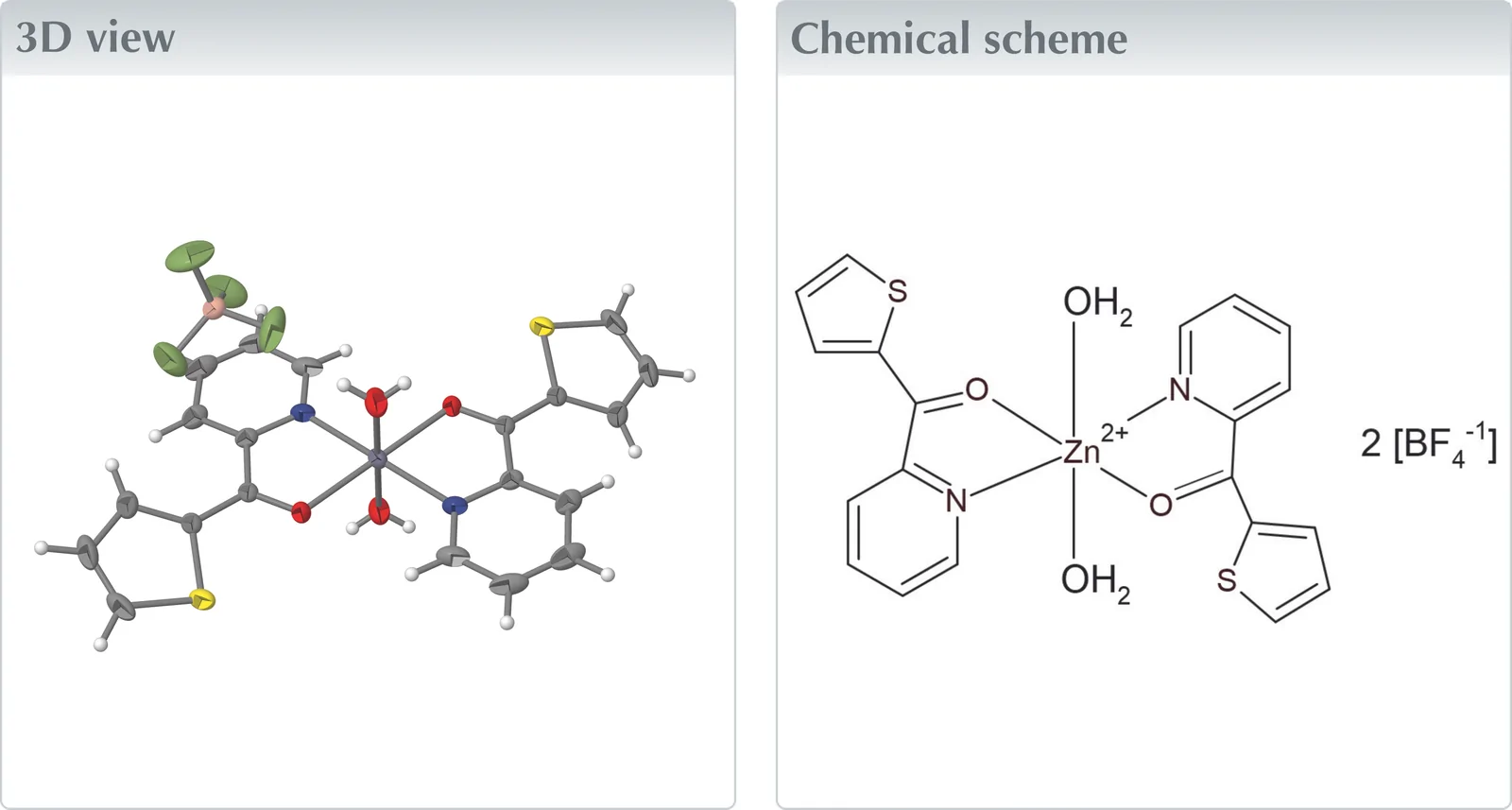

## Structure description

**Figure 1:**
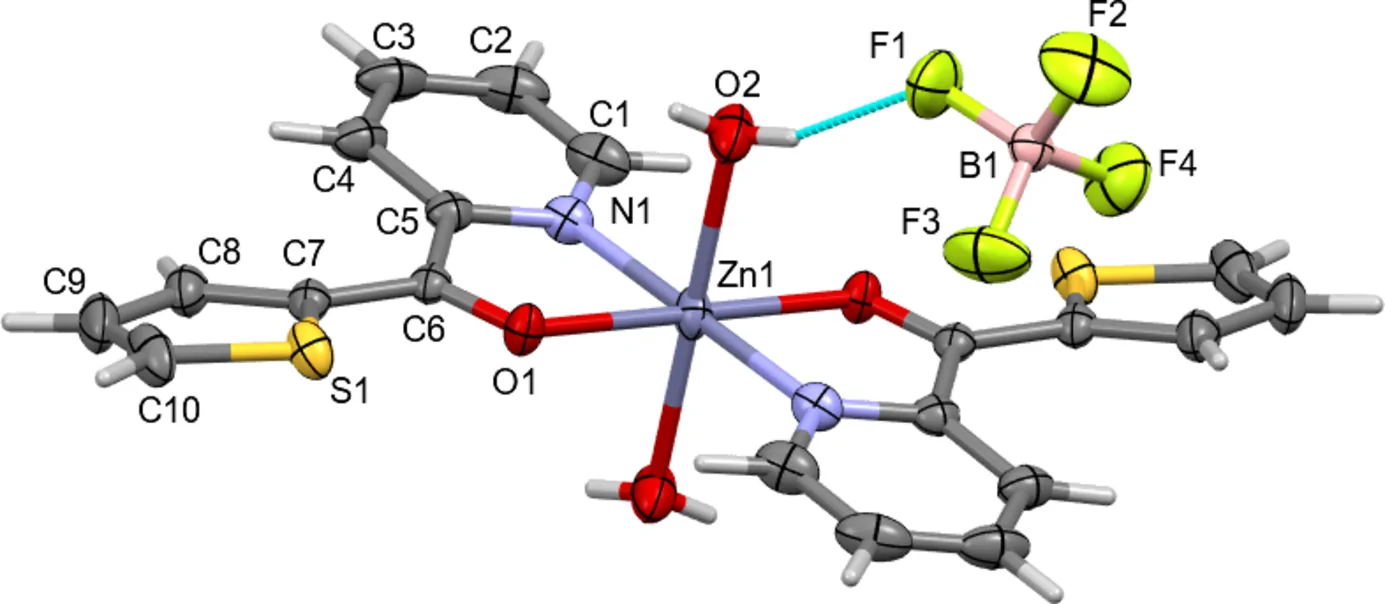
The mol­ecular entities in the asymmetric unit of the title salt shown with displacement ellipsoids at the 50% probability level. O—H⋯F hy­dro­gen bonding is shown by a dashed line. Atoms of the asymmetric unit are labeled and non-labeled atoms are generated by the symmetry code (−*x* + 1, −*y* + 1, −*z* + 1).

The com­plex title salt (Fig. 1 ▸) is structurally similar to the previously reported Cu^II^ (Sommerer *et al.*, 1998 ▸) and Ni^II^ (Westcott & Nichol, 2026 ▸) analogs. It is isotypic with the Co^II^ com­plex (Westcott, 2026 ▸), but unlike that crystal structure, it has an ordered arrangement of the thio­phene units. The title salt also resembles other Zn^II^ com­plexes with related ligands, such as di-2-pyridyl ketone (Kanaras *et al.*, 2004 ▸) and di-2-pyridyl ketone oxime (Westcott *et al.*, 2016 ▸).

The zinc atom in the title compound is located at an inversion center with pseudo-octa­hedral coordination. The 2-thienyl 2-pyridyl ketone (tpk) ligands coordinate through the pyridyl N [2.0624 (18) Å] and carbonyl O [2.1030 (13) Å] atoms in the equatorial plane, and the O atoms from water mol­ecules coordinate in the axial positions, leading to an [O_4_N_2_] coordination set. The Zn—O distance with the aqua ligands [2.1598 (16) Å] is within the distances seen for the previous three com­plexes, as are all other bond lengths and angles. The thienyl ring in the title com­plex is 20.78 (8)° out of the plane defined by the Zn^II^ site and the pyridyl ring, consistent with what is seen for Co^II^ [21.22 (8)°] (Westcott, 2026 ▸) and Ni^II^ [19.01 (7)°] (Westcott & Nichol, 2026 ▸).

**Figure 2:**
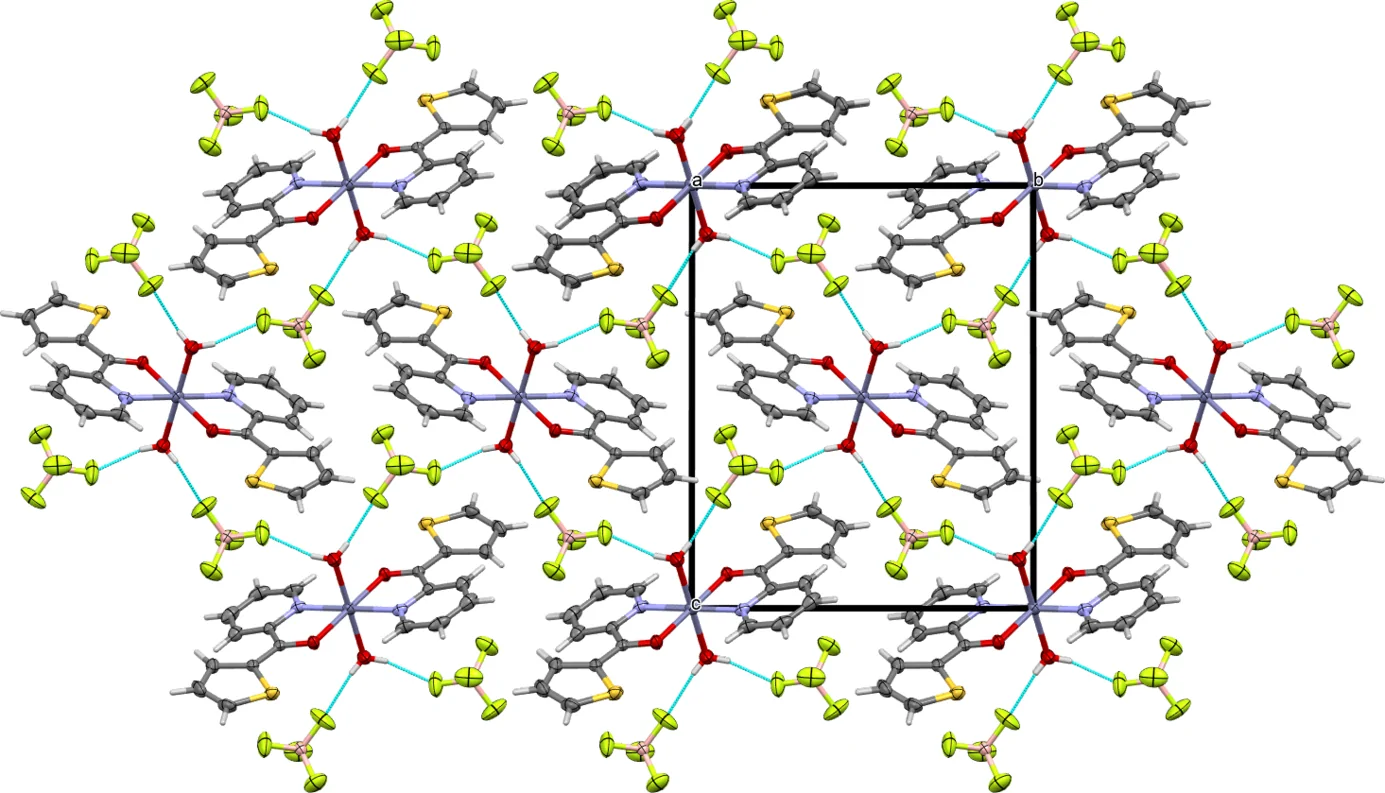
The crystal structure of the title salt viewed along the *a*-axis direction. O—H⋯F hy­dro­gen bonding is shown by dashed lines.

The coordinating water mol­ecules are hy­dro­gen bonded to the BF_4_^−^ anion (Table 1 ▸), forming layers extending parallel to (10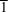) (Fig. 2 ▸).

## Synthesis and crystallization

Zn(BF_4_)_2_·H_2_O and aceto­nitrile were used as received from Thermo Fisher, and di-2-pyridyl ketone (tpk) was used as received from Rieke Metals. The title com­plex was synthesized following a literature procedure (Sommerer *et al.*, 1998 ▸). Excess Zn(BF_4_)_2_·6H_2_O (0.2318 g, 0.9019 mmol) was combined with tpk (0.3237 g, 1.711 mmol) in 40 ml of aceto­nitrile at room tem­per­a­ture, affording a colorless solution, which was allowed to evaporate slowly to facilitate the formation of yellow crystals suitable for X-ray diffraction (18 d).

## Refinement

Crystal data, data collection, and structure refinement details are summarized in Table 2 ▸. Water H-atom positions were refined maintaining a tetra­hedral Zn—O—H angle and allowing O—H distances to obtain the best fit to the observed electron density.

## Supplementary Material

Crystal structure: contains datablock(s) I. DOI: 10.1107/S2414314626010369/wm4262sup1.cif

Structure factors: contains datablock(s) I. DOI: 10.1107/S2414314626010369/wm4262Isup2.hkl

CCDC reference: 2593362

Additional supporting information:  crystallographic information; 3D view; checkCIF report

## Figures and Tables

**Table 1:** Hydrogen-bond geometry (Å, °)

*D*—H⋯*A*	*D*—H	H⋯*A*	*D*⋯*A*	*D*—H⋯*A*
O2—H2*A*⋯F1	0.76	1.97	2.691 (2)	160
O2—H2*B*⋯F2^i^	0.71	2.01	2.709 (2)	171

Symmetry code: (i) 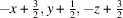.

**Table 2:** Experimental details

Crystal data	
Chemical formula	[Zn(C_10_H_7_BF_4_NOS)_2_(H_2_O)_2_](BF_4_)_2_
*M*_r_	653.47
Crystal system, space group	Monoclinic, *P*2_1_/*n*
Temperature (K)	120
*a*, *b*, *c* (Å)	7.1601 (3), 11.9574 (4), 15.2293 (5)
β (°)	103.519 (2)
*V* (Å^3^)	1267.75 (8)
*Z*	2
Radiation type	Cu *K*α
μ (mm^−1^)	3.73
Crystal size (mm)	0.16 × 0.15 × 0.06

Data collection	
Diffractometer	Bruker Venture
Absorption correction	Numerical (*SADABS*; Krause *et al.*, 2015 ▸)
*T*_min_, *T*_max_	0.632, 0.792
No. of measured, independent and observed [*I* > 2σ(*I*)] reflections	50901, 2591, 2426
*R*_int_	0.040
(sin θ/λ)_max_ (Å^−1^)	0.625

Refinement	
*R*[*F*^2^ > 2σ(*F*^2^)], *wR*(*F*^2^), *S*	0.033, 0.085, 1.05
No. of reflections	2591
No. of parameters	182
H-atom treatment	H atoms treated by a mixture of independent and constrained refinement
Δρ_max_, Δρ_min_ (e Å^−3^)	0.90, −0.44

Computer programs: *APEX5* (Bruker, 2023 ▸), *SAINT* (Bruker, 2023 ▸), *SHELXT* (Sheldrick, 2015*a* ▸), *SHELXL* (Sheldrick, 2015*b* ▸), *ShelXle* (Hübschle *et al.*, 2011 ▸), *Mercury* (Macrae *et al.*, 2020 ▸), and *publCIF* (Westrip, 2010 ▸).

## full crystallographic data

### trans-Diaquabis(pyridin-2-yl thiophen-2-yl ketone-κ2N,O)zinc(II) bis(tetrafluoridoborate) Crystal data

**Table:**

[Zn(C_10_H_7_BF_4_NOS)_2_(H_2_O)_2_](BF_4_)_2_	*F*(000) = 656
*M**_r_* = 653.47	*D*_x_ = 1.712 Mg m^−^^3^
Monoclinic, *P*2_1_/*n*	Cu *K*α radiation, λ = 1.54178 Å
*a* = 7.1601 (3) Å	Cell parameters from 9706 reflections
*b* = 11.9574 (4) Å	θ = 4.8–74.6°
*c* = 15.2293 (5) Å	µ = 3.73 mm^−^^1^
β = 103.519 (2)°	*T* = 120 K
*V* = 1267.75 (8) Å^3^	Block, colourless
*Z* = 2	0.16 × 0.15 × 0.06 mm

### trans-Diaquabis(pyridin-2-yl thiophen-2-yl ketone-κ2N,O)zinc(II) bis(tetrafluoridoborate) Data collection

**Table:**

Bruker Venture diffractometer	2591 independent reflections
Radiation source: micro-focus, Incoatec Diamond	2426 reflections with *I* > 2σ(*I*)
HELIOS Multi-layer monochromator	*R*_int_ = 0.040
Detector resolution: 7.41 pixels mm^-1^	θ_max_ = 74.6°, θ_min_ = 4.8°
combination of ω and φ–scans	*h* = −8→8
Absorption correction: numerical (*SADABS*; Krause *et al.*, 2015)	*k* = −14→14
*T*_min_ = 0.632, *T*_max_ = 0.792	*l* = −18→19
50901 measured reflections	

### trans-Diaquabis(pyridin-2-yl thiophen-2-yl ketone-κ2N,O)zinc(II) bis(tetrafluoridoborate) Refinement

**Table:**

Refinement on *F*^2^	0 restraints
Least-squares matrix: full	Hydrogen site location: inferred from neighbouring sites
*R*[*F*^2^ > 2σ(*F*^2^)] = 0.033	H atoms treated by a mixture of independent and constrained refinement
*wR*(*F*^2^) = 0.085	*w* = 1/[σ^2^(*F*_o_^2^) + (0.0365*P*)^2^ + 1.6051*P*] where *P* = (*F*_o_^2^ + 2*F*_c_^2^)/3
*S* = 1.05	(Δ/σ)_max_ < 0.001
2591 reflections	Δρ_max_ = 0.90 e Å^−^^3^
182 parameters	Δρ_min_ = −0.44 e Å^−^^3^

### trans-Diaquabis(pyridin-2-yl thiophen-2-yl ketone-κ2N,O)zinc(II) bis(tetrafluoridoborate) Special details

**Table:**

Geometry. All esds (except the esd in the dihedral angle between two l.s. planes) are estimated using the full covariance matrix. The cell esds are taken into account individually in the estimation of esds in distances, angles and torsion angles; correlations between esds in cell parameters are only used when they are defined by crystal symmetry. An approximate (isotropic) treatment of cell esds is used for estimating esds involving l.s. planes.

### trans-Diaquabis(pyridin-2-yl thiophen-2-yl ketone-κ2N,O)zinc(II) bis(tetrafluoridoborate) Fractional atomic coordinates and isotropic or equivalent isotropic displacement parameters (Å^2^)

**Table:**

	*x*	*y*	*z*	*U*_iso_*/*U*_eq_	
Zn1	0.500000	0.500000	0.500000	0.02437 (12)	
S1	0.18759 (8)	0.72375 (5)	0.69959 (3)	0.03012 (14)	
F1	0.8022 (3)	0.24775 (13)	0.67903 (14)	0.0624 (5)	
F2	0.8089 (3)	0.07751 (18)	0.73899 (13)	0.0714 (6)	
F3	0.5264 (2)	0.15039 (18)	0.66175 (11)	0.0608 (5)	
F4	0.7480 (3)	0.09858 (16)	0.58875 (11)	0.0579 (5)	
C1	0.7890 (3)	0.6652 (2)	0.45821 (15)	0.0344 (5)	
H1	0.826771	0.605726	0.424795	0.041*	
O1	0.3691 (2)	0.60322 (11)	0.58006 (9)	0.0244 (3)	
O2	0.7274 (3)	0.45695 (15)	0.61534 (11)	0.0389 (4)	
H2A	0.725 (2)	0.395 (2)	0.6240 (11)	0.052 (10)*	
H2B	0.724 (2)	0.4932 (17)	0.6516 (13)	0.068 (13)*	
N1	0.6421 (2)	0.64948 (15)	0.49702 (11)	0.0252 (4)	
C2	0.8881 (3)	0.7654 (2)	0.46508 (16)	0.0397 (6)	
H2	0.989151	0.775495	0.435059	0.048*	
C3	0.8378 (3)	0.8496 (2)	0.51593 (17)	0.0395 (6)	
H3	0.904055	0.919023	0.521513	0.047*	
C4	0.6892 (3)	0.83319 (19)	0.55943 (15)	0.0310 (5)	
H4	0.656277	0.889617	0.597064	0.037*	
C5	0.5907 (3)	0.73282 (17)	0.54654 (13)	0.0232 (4)	
C6	0.4250 (3)	0.70198 (16)	0.58704 (12)	0.0207 (4)	
C7	0.3300 (3)	0.78074 (16)	0.63380 (13)	0.0230 (4)	
C8	0.3224 (3)	0.89730 (18)	0.63450 (15)	0.0315 (5)	
H8	0.390280	0.944439	0.602466	0.038*	
C9	0.2018 (4)	0.9361 (2)	0.68855 (17)	0.0418 (6)	
H9	0.178309	1.013201	0.696706	0.050*	
C10	0.1221 (4)	0.8528 (2)	0.72797 (15)	0.0400 (6)	
H10	0.038714	0.865221	0.767203	0.048*	
B1	0.7191 (3)	0.14287 (19)	0.66790 (16)	0.0245 (5)	

### trans-Diaquabis(pyridin-2-yl thiophen-2-yl ketone-κ2N,O)zinc(II) bis(tetrafluoridoborate) Atomic displacement parameters (Å^2^)

**Table:**

	*U*^11^	*U*^22^	*U*^33^	*U*^12^	*U*^13^	*U*^23^
Zn1	0.0334 (2)	0.0206 (2)	0.0211 (2)	0.00279 (15)	0.01042 (15)	−0.00211 (14)
S1	0.0370 (3)	0.0322 (3)	0.0241 (3)	0.0106 (2)	0.0131 (2)	0.00448 (19)
F1	0.0648 (11)	0.0253 (7)	0.0870 (13)	−0.0074 (7)	−0.0029 (9)	−0.0029 (8)
F2	0.0715 (12)	0.0805 (14)	0.0575 (11)	0.0102 (10)	0.0054 (9)	0.0448 (10)
F3	0.0303 (8)	0.1036 (15)	0.0507 (9)	0.0004 (8)	0.0138 (7)	−0.0034 (9)
F4	0.0630 (10)	0.0683 (11)	0.0479 (9)	−0.0027 (9)	0.0242 (8)	−0.0216 (8)
C1	0.0295 (11)	0.0502 (14)	0.0257 (10)	0.0037 (10)	0.0110 (9)	0.0086 (10)
O1	0.0304 (7)	0.0194 (7)	0.0265 (7)	−0.0024 (5)	0.0131 (6)	−0.0030 (5)
O2	0.0571 (11)	0.0268 (9)	0.0273 (8)	0.0076 (8)	−0.0009 (7)	−0.0034 (7)
N1	0.0262 (8)	0.0301 (9)	0.0205 (8)	0.0013 (7)	0.0083 (7)	0.0042 (7)
C2	0.0251 (11)	0.0616 (16)	0.0326 (12)	−0.0038 (11)	0.0071 (9)	0.0184 (11)
C3	0.0273 (11)	0.0456 (14)	0.0408 (13)	−0.0125 (10)	−0.0018 (10)	0.0167 (11)
C4	0.0277 (11)	0.0302 (11)	0.0317 (11)	−0.0055 (9)	0.0004 (8)	0.0057 (9)
C5	0.0237 (9)	0.0245 (10)	0.0199 (9)	−0.0003 (8)	0.0017 (7)	0.0047 (7)
C6	0.0238 (9)	0.0203 (9)	0.0162 (8)	0.0008 (7)	0.0012 (7)	0.0003 (7)
C7	0.0280 (10)	0.0215 (10)	0.0180 (9)	0.0019 (7)	0.0026 (7)	−0.0001 (7)
C8	0.0376 (12)	0.0233 (10)	0.0304 (11)	0.0049 (9)	0.0019 (9)	−0.0013 (8)
C9	0.0525 (15)	0.0299 (12)	0.0383 (13)	0.0132 (11)	0.0013 (11)	−0.0125 (10)
C10	0.0493 (14)	0.0466 (14)	0.0244 (11)	0.0210 (11)	0.0092 (10)	−0.0058 (10)
B1	0.0264 (11)	0.0221 (11)	0.0249 (11)	0.0003 (9)	0.0059 (9)	0.0031 (9)

### trans-Diaquabis(pyridin-2-yl thiophen-2-yl ketone-κ2N,O)zinc(II) bis(tetrafluoridoborate) Geometric parameters (Å, º)

**Table:**

Zn1—N1^i^	2.0624 (18)	O2—H2B	0.71 (2)
Zn1—N1	2.0624 (18)	N1—C5	1.352 (3)
Zn1—O1^i^	2.1030 (13)	C2—C3	1.369 (4)
Zn1—O1	2.1030 (13)	C2—H2	0.9500
Zn1—O2	2.1598 (16)	C3—C4	1.392 (3)
Zn1—O2^i^	2.1598 (16)	C3—H3	0.9500
S1—C10	1.697 (2)	C4—C5	1.383 (3)
S1—C7	1.728 (2)	C4—H4	0.9500
F1—B1	1.381 (3)	C5—C6	1.506 (3)
F2—B1	1.368 (3)	C6—C7	1.443 (3)
F3—B1	1.364 (3)	C7—C8	1.395 (3)
F4—B1	1.376 (3)	C8—C9	1.404 (3)
C1—N1	1.335 (3)	C8—H8	0.9500
C1—C2	1.383 (4)	C9—C10	1.357 (4)
C1—H1	0.9500	C9—H9	0.9500
O1—C6	1.243 (2)	C10—H10	0.9500
O2—H2A	0.76 (2)		
			
N1^i^—Zn1—N1	180.0	C2—C3—H3	120.1
N1^i^—Zn1—O1^i^	78.06 (6)	C4—C3—H3	120.1
N1—Zn1—O1^i^	101.94 (6)	C5—C4—C3	118.4 (2)
N1^i^—Zn1—O1	101.94 (6)	C5—C4—H4	120.8
N1—Zn1—O1	78.06 (6)	C3—C4—H4	120.8
O1^i^—Zn1—O1	180.00 (6)	N1—C5—C4	121.54 (19)
N1^i^—Zn1—O2	93.30 (7)	N1—C5—C6	113.04 (17)
N1—Zn1—O2	86.70 (7)	C4—C5—C6	125.39 (19)
O1^i^—Zn1—O2	88.75 (6)	O1—C6—C7	118.84 (18)
O1—Zn1—O2	91.25 (6)	O1—C6—C5	117.80 (17)
N1^i^—Zn1—O2^i^	86.70 (7)	C7—C6—C5	123.35 (17)
N1—Zn1—O2^i^	93.30 (7)	C8—C7—C6	132.8 (2)
O1^i^—Zn1—O2^i^	91.25 (6)	C8—C7—S1	111.11 (16)
O1—Zn1—O2^i^	88.75 (6)	C6—C7—S1	116.03 (15)
O2—Zn1—O2^i^	180.0	C7—C8—C9	111.4 (2)
C10—S1—C7	91.42 (12)	C7—C8—H8	124.3
N1—C1—C2	122.1 (2)	C9—C8—H8	124.3
N1—C1—H1	118.9	C10—C9—C8	113.4 (2)
C2—C1—H1	118.9	C10—C9—H9	123.3
C6—O1—Zn1	115.38 (12)	C8—C9—H9	123.3
Zn1—O2—H2A	109.5	C9—C10—S1	112.67 (18)
Zn1—O2—H2B	109.5	C9—C10—H10	123.7
H2A—O2—H2B	117.4	S1—C10—H10	123.7
C1—N1—C5	119.2 (2)	F3—B1—F2	111.9 (2)
C1—N1—Zn1	125.16 (16)	F3—B1—F4	108.78 (19)
C5—N1—Zn1	115.26 (13)	F2—B1—F4	109.5 (2)
C3—C2—C1	118.8 (2)	F3—B1—F1	110.4 (2)
C3—C2—H2	120.6	F2—B1—F1	108.2 (2)
C1—C2—H2	120.6	F4—B1—F1	108.0 (2)
C2—C3—C4	119.8 (2)		

Symmetry code: (i) −*x*+1, −*y*+1, −*z*+1.

### trans-Diaquabis(pyridin-2-yl thiophen-2-yl ketone-κ2N,O)zinc(II) bis(tetrafluoridoborate) Hydrogen-bond geometry (Å, º)

**Table:**

*D*—H···*A*	*D*—H	H···*A*	*D*···*A*	*D*—H···*A*
O2—H2*A*···F1	0.76	1.97	2.691 (2)	160
O2—H2*B*···F2^ii^	0.71	2.01	2.709 (2)	171

Symmetry code: (ii) −*x*+3/2, *y*+1/2, −*z*+3/2.
